# Characterization of Skilled Nursing Facility Residents Admitted With Substance Use Disorders

**DOI:** 10.1093/geroni/igaa057.143

**Published:** 2020-12-16

**Authors:** Rhea Sindvani, Lisa Barry

**Affiliations:** 1 UConn Health, Farmington, Connecticut, United States; 2 UConn Center on Aging, Farmington, Connecticut, United States

## Abstract

For reasons including the opioid epidemic and more widespread substance use across all ages, the prevalence of individuals meeting preadmission criteria for skilled nursing facility (SNF)-level care and who have substance use disorders (SUDs) is growing. However, little is known about this population. We characterized a sample of residents with SUDs in two SNFs that target admission of difficult-to-place individuals in Hartford, Connecticut. Residents admitted between June 1, 2018 and May 31, 2019 and had an SUD per Pre-Admission Screening and Resident Review (PASRR) were included. Using retrospective chart review, we collected data including demographics, physical and mental health conditions, psychiatric medications, and participation in SNF-provided SUD counseling. Of 163 residents admitted with an SUD, all were admitted following an acute hospitalization. Residents’ average age was 49.9(SD=11.7) years (range 21-79). They were 61% male and racially diverse; 56% Caucasian, 27% Hispanic, 16% Black. SUDs on admission included opioid use disorder (48%), alcohol use disorder (33%), unspecified psychoactive SUD (26%), cocaine use disorder (25%), and Other (20%). Of these, 18% and 16% were taking methadone or suboxone, respectively and 25% were taking an antipsychotic medication. Comorbidities such as bipolar disorder (15%) and viral hepatitis (26%) were prevalent. A total of 40 (25%) residents participated in SUD counseling; none of the aforementioned factors was associated with participation. This is the first study to characterize a sample of residents from SNFs that target individuals with SUDs. Improved understanding of this unique and growing subset of the SNF population may help optimize their treatment.

